# Constructing a competency model for family education guidance among rural teachers in ethnic minority border regions: a grounded theory approach

**DOI:** 10.3389/fpsyg.2026.1772630

**Published:** 2026-07-06

**Authors:** Qiying Gan, Hong Wang

**Affiliations:** Faculty of Teacher Education, Wenshan University, Wenshan, China

**Keywords:** ethnic minority border regions, family education guidance competency, grounded theory, rural teachers, Southwest China, teacher professional development, Yunnan Province

## Abstract

Rural families residing in ethnic minority border regions require precisely targeted family education guidance, and a professionally trained teaching workforce constitutes a critical prerequisite for the successful implementation of school-based family education guidance services. Employing a qualitative research paradigm grounded in constructivist epistemology, this study investigates the competency structure underlying family education guidance among rural teachers operating within ethnic minority border regions. Utilizing grounded theory methodology, data were collected through semi-structured interviews with 25 teachers and subsequently analyzed through a rigorous three-level coding procedure. The findings reveal that the family education guidance competency of rural teachers in ethnic minority border regions comprises five interconnected core categories: (1) knowledge and professional competency, (2) communication and interaction competency, (3) cross-cultural and multilingual competency, (4) guidance and motivation competency, and (5) psychological support and management competency. These competencies demonstrate significant interrelationships, collectively forming a comprehensive family education guidance competency model specifically tailored to rural teachers in ethnic minority border regions. The theoretical model developed in this study effectively addresses the distinctive educational environment characteristic of ethnic minority border regions, responds to the specialized needs of rural teachers engaged in family education guidance, and provides both theoretical foundations and practical guidance for enhancing teachers' family education guidance competency in these underserved contexts.

## 1 Introduction

Teacher competency in family education guidance has emerged as a critical research domain within the broader field of teacher professional development. Although quantitative approaches have long dominated investigations of parental involvement and its relationship to student outcomes, qualitative methodologies offer distinctive advantages for capturing the nuanced, context-specific competencies that teachers draw upon in diverse educational settings (Creswell and Poth, 2018). For example, a meta-synthesis of parental involvement research—though conducted primarily within a quantitative tradition—highlighted the need for deeper exploration of the mechanisms through which teachers facilitate effective family–school partnerships (Wilder, 2014). Findings of this kind have prompted scholars to adopt interpretive research paradigms capable of representing the complexity of teacher–parent interactions in culturally diverse contexts. Yet the turn toward interpretive inquiry does not lessen the need for theoretical grounding; it instead foregrounds a prior question: which conceptual frameworks can adequately inform research on family education guidance in such settings? The theoretical foundations for addressing this question draw upon multiple disciplinary perspectives. An influential model of overlapping school, family, and community spheres provides a structural foundation for conceptualizing partnership (Epstein, 2011), yet its application in non-Western and rural contexts requires substantial adaptation. At the level of professional policy, standards frameworks such as Quebec's Reference Framework for Professional Competencies for Teachers have formally recognized cooperation with families as a core teaching competency (Antony-Newman, 2024). Such frameworks, however, predominantly reflect urban, mainstream educational contexts, and they may inadequately address the specialized competencies demanded in ethnically and linguistically diverse rural settings. Empirical research on teacher competencies for parental cooperation has further established that effective family education guidance requires specialized professional skills extending beyond general pedagogical knowledge, with teachers needing distinct competencies to navigate the interpersonal dynamics inherent in parent–teacher relationships (Westergård, 2013). Nevertheless, existing competency frameworks have been developed largely through deductive approaches that apply predetermined categories to diverse populations, rather than inductively deriving competency structures from practitioners' lived experiences. These limitations are particularly consequential in ethnic minority border regions,^1^ whose educational challenges call for context-sensitive frameworks grounded in local knowledge and practice. Such regions present a confluence of factors—geographic isolation, cultural plurality, linguistic diversity, and resource constraints—that distinguishes them from both urban centers and homogeneous rural communities. Teachers working in these environments must pursue the universal objectives of family education guidance while continually adapting their practice to the distinctive circumstances of ethnic minority communities. A seminal synthesis of research on family–school connections compellingly established the relationship between family engagement and student achievement, yet it was developed largely within mainstream, English-speaking contexts and did not address the cross-cultural and multilingual dynamics central to effective practice in multiethnic borderland settings (Henderson and Mapp, 2002). The present study takes this synthesis as its point of departure and seeks to extend it to the competencies that ethnic minority border contexts specifically demand—an aspect it left largely unexamined. To establish the conceptual basis for this inquiry, the following section reviews existing scholarship on the dimensions of teachers' family education guidance competency.

## 2 Literature review

### 2.1 Dimensions of teachers' family education guidance competency

Teachers' family education guidance competency is best understood as a multidimensional construct that integrates cognitive, communicative, emotional and ethical, organizational, and developmental capacities. Drawing on the existing literature, these capacities can be grouped into several interrelated dimensions.

A foundational dimension concerns teachers' theoretical knowledge and professional understanding of family engagement. Scholars of teacher preparation have long argued that effective partnership work rests on a specialized knowledge base that initial teacher education has tended to neglect, leaving many teachers to enter practice without firm conceptual grounding (Willemse et al., 2018). Part of this knowledge base consists of theoretical accounts of how and why families become involved in their children's education (Hoover-Dempsey and Sandler, 1997), together with a professional identification with family engagement as a legitimate dimension of the teaching role rather than a peripheral duty (Antony-Newman, 2024).

Second, communication and collaboration constitute central competencies. In an interview study of teachers' cooperation with parents, Westergård (2013) identified relational and communicative competencies—together with a “context competence” attuned to the school's particular circumstances—as decisive for productive partnerships. In the Chinese context, home–school cooperation carries a distinctive educational value shaped by the complementary yet differentiated roles of family and school, and grasping this value is a prerequisite for substantive cooperation in practice (Huang and Ma, 2011). Epstein's (2011) theory of overlapping spheres of influence has likewise been synthesized within Chinese conditions, showing how family, school, and community spheres intersect in locally specific ways—a point of particular relevance for ethnically and linguistically diverse rural communities, where these spheres may overlap in non-standard configurations (Zhang et al., 2019). Effective family education guidance accordingly requires teachers to understand families' perspectives and to coordinate with the various actors involved in a child's education.

Third, emotional and ethical qualities are indispensable. Goodall and Montgomery (2014) distinguished a continuum extending from parental involvement toward genuine parental engagement, arguing that movement toward the more agentic end depends on relationships of trust and on teachers' affective investment. Ethical commitments—prioritizing children's developmental interests and positioning parents as partners rather than as passive recipients of advice—are likewise fundamental to guidance work.

Fourth, organizational and managerial competencies allow teachers to translate good intentions into sustained practice. Because teacher education has historically devoted limited systematic attention to family engagement (Willemse et al., 2018; Antony-Newman, 2024), many teachers draw on accumulated personal experience in the absence of professional training—an observation that underscores the need to develop teachers' capacity to design and organize activities, provide individualized guidance, and engage in reflective practice.

Fifth, professional skills and ongoing development warrant attention. Sustaining effective guidance over time requires that teachers continue to expand their professional repertoire and exercise practical, adaptive judgment when confronting the diverse and frequently unpredictable situations that family education guidance presents; the “context competence” highlighted by Westergård (2013) captures precisely this capacity to read and respond to particular circumstances.

### 2.2 Strategies for enhancing teachers' family education guidance competency

Scholarship on how to strengthen this competency converges on three broad strategies: cultivating teachers' awareness and professional values, providing practice-oriented preparation and support, and building collaborative and institutional structures.

First, enhancement begins with teachers' beliefs and professional values. Whether teachers construe family engagement as integral to their role—rather than as an externally assigned task—shapes whether and how they pursue it; the concept of role construction (Hoover-Dempsey and Sandler, 1997) captures this orientation, and critical policy analysis indicates that the formal recognition of engagement as a core professional competency is itself a precondition for cultivating teacher readiness (Antony-Newman, 2024).

Second, systematic, practice-oriented preparation is widely advocated as a remedy for teachers' reliance on personal experience. Teacher-education scholars call for embedding family-engagement content within initial teacher education and continuing professional development through participatory, experiential, and case-based approaches (Willemse et al., 2016, 2018). Manageable workloads and institutional support are, in turn, regarded as enabling conditions that allow such preparation to translate into practice.

Third, durable improvement depends on collaborative and institutional structures. Epstein's (2011) framework of overlapping school, family, and community spheres provides the canonical model for organizing programs that integrate the complementary contributions of these settings, while a supportive institutional culture—clear professional standards, defined responsibilities, appropriate incentives, and accountability mechanisms—offers the conditions under which teachers' family education guidance can be sustained.

Importantly, the difficulty of supporting family engagement across cultural, linguistic, and geographic divides is not unique to the present setting, and a substantial body of work in adjacent contexts offers valuable orientation. In the U.S.–Mexico borderlands, Valdés's (1996) 3-year ethnography of Mexican immigrant families showed that home–school distance reflected a mismatch between schools' mainstream assumptions and families' own values and cultural capital rather than parental indifference, and it cautioned that interventions disregarding those values may prove counterproductive. A parallel line of inquiry in rural special education has examined how families in geographically isolated and culturally diverse communities engage with schools: Burke (2017) found that rural Latino families of children with disabilities encountered context-bound barriers to advocacy—such as discrimination—distinct from the disability-related barriers reported by their urban counterparts, while Buren et al. (2022) documented how limited local resources constrained rural families' capacity to partner with schools and exacted an emotional toll on those sustaining such relationships.

The present study is situated within general education and therefore does not address the specialized legal and procedural dimensions—such as individualized education planning—that structure family engagement in special education; this special-education scholarship is drawn upon not as the study's object but as a source of transferable insight into rural and culturally diverse family–school partnerships. These international parallels are instructive, yet they cannot substitute for engagement with the literature on China's own minority border regions, to which the discussion now turns.

### 2.3 Family education and schooling in the ethnic minority border regions of Southwest China

The preceding strands of scholarship, developed largely in North American, European, or Han-majority urban contexts, must be read alongside a substantial body of Chinese research on ethnic minority education (minzu jiaoyu) that speaks directly to the conditions of Southwest China. Foundational work establishing the disciplinary system of ethnic education studies in China conceptualizes minority schooling as inseparable from bilingual instruction, multicultural curriculum, and the negotiation of cultural difference—dimensions that remain peripheral in mainstream family–school partnership models but are central in the present context (Wang, 2003). Comparative scholarship further demonstrates that frameworks imported from Western multicultural education cannot be applied wholesale to China's minority regions without attending to the specific historical and policy environment in which Chinese minority education is embedded (Wang and Wan, 2008). The theory of multicultural integration education provides a particularly useful lens, holding that effective education in multiethnic settings must simultaneously transmit the national common culture and affirm the distinct cultural heritage of each ethnic group—a dual imperative that border-region teachers must negotiate in virtually every encounter with families (Li and Teng, 2020).

A second strand of this literature concerns the linguistic dimension of minority schooling. Analyses of bilingual education policy document the persistent structural tension between instruction in the national language and the maintenance of ethnic mother tongues, a tension that becomes acute at the level of home–school communication when parents are functionally monolingual in an ethnic language (Wan and Liu, 2012). This body of work also helps explain the historically rooted mistrust that some minority families hold toward state schooling: earlier assimilationist phases of language policy, under which the use of ethnic languages in school was actively discouraged, left a legacy of wariness that contemporary teachers must work patiently to overcome. Ethnographic research on bilingual and bicultural education in Southwest China similarly establishes that the cultural and linguistic distance between minority families and the Han-majority school system generates barriers to family–school communication that are qualitatively different from those documented in mainstream rural or urban settings (Teng, 2001).

A third strand addresses the dynamics specific to Yunnan's international borderlands. Reviews of cross-border ethnic education identify the distinctive challenges that arise where ethnic communities span national boundaries, including the movement of students and families across the borders with Myanmar, Laos, and Vietnam and the resulting entanglement of educational decisions with cross-border economic and kinship networks (He, 2009). Subsequent work has framed these challenges in terms of cultural security and national identity, underscoring that schooling in the border region carries social and political weight largely absent from interior contexts (He, 2010), while more recent scholarship has examined how the Belt and Road Initiative has intensified cross-border educational mobility and the demands it places on frontline teachers (Chen and Liu, 2020). Compounding these border dynamics is the prevalence of left-behind children: large-scale empirical research has shown that the migration of parents to distant urban labor markets leaves children in the care of grandparents who are frequently unable to provide the educationally supportive home environment that schools assume, a problem especially concentrated in poor border counties (Duan et al., 2014; Wan and Mao, 2010). Taken together, this Chinese-language scholarship richly documents the historical, linguistic, cross-border, and socioeconomic conditions of education in Southwest China's minority border regions. What it has not yet produced, however, is an empirically grounded model of the specific competencies that rural teachers require to deliver family education guidance under these conditions; existing work has likewise paid limited attention to how variation in family background and the distinctive characteristics of this teacher population shape the formation of such competency. It is this gap—between a well-developed contextual literature and the absence of a context-specific teacher-competency framework—that the present study sets out to address through an inductive, qualitative investigation grounded in practitioners' own experience.

## 3 Research design

### 3.1 Research methodology

This study adopted a grounded theory approach to achieve an in-depth understanding and systematic interpretation of family education guidance competency among rural teachers in ethnic minority border regions. Grounded theory is a rigorous qualitative methodology that enables researchers to extract meaningful patterns from richly detailed data collected within specific cultural contexts, thereby facilitating deep exploration of participants' perspectives and lived experiences (Corbin and Strauss, 2015; Charmaz, 2014).

Given the complexity and distinctiveness of rural teachers' work in ethnic minority border regions within the domain of family education guidance, this study employed grounded theory to inductively develop theoretical constructs from interview data, thereby revealing the practical difficulties and challenges these teachers encounter throughout the family education guidance process. Through systematic interview protocols and three-level coding analysis (open, axial, and selective coding), the research sought to comprehensively capture the challenges faced by rural teachers, the adaptive strategies they employ, and their expressed needs for competency enhancement. The grounded theory methodology allowed researchers to explore participants' personal experiences and perspectives in depth, distilling key insights and providing empirically grounded support and recommendations for improving family education guidance competency among rural teachers in ethnic minority border regions (Stough and Lee, 2021).

The choice of grounded theory over alternative qualitative approaches warrants explicit justification. Narrative inquiry, which foregrounds individuals' storied accounts, and phenomenological approaches, which seek to distill the essence of a shared lived experience, would have brought certain complementary strengths to this inquiry. However, the primary aim of the present study was not to represent the lived experience of a single teacher or a small group in its full biographical depth, but rather to construct an integrated, transferable competency model grounded in the collective experiences of a purposively diverse sample. Grounded theory's systematic comparative coding—moving from open coding through axial coding to selective coding—is specifically designed to generate mid-range theoretical frameworks from empirical data (Corbin and Strauss, 2015; Charmaz, 2014). At the same time, the constructivist variant adopted here retains a commitment to foregrounding participants' own voices through extensive verbatim quotation, ensuring that the resulting model remains anchored in the language and meaning-making of the teachers themselves rather than being imposed from outside.

This study was conducted in accordance with the ethical principles for research involving human participants set out in the Declaration of Helsinki. The research protocol was reviewed and approved by the Academic Ethics Committee of Wenshan University (approval no. 20230019).

### 3.2 Participants

The ethnic minority border regions of Yunnan Province encompass eight border prefectures and twenty-five border counties. Employing a maximum-variation purposive sampling strategy, a total of 25 primary and secondary school teachers were selected to participate in semi-structured interviews. Participants were chosen to satisfy five criteria: (1) current frontline teaching in a rural school located in one of Yunnan's border prefectures; (2) breadth of ethnic background—the final sample spans Zhuang, Hani, Yao, Miao, Lisu, Dai, Jingpo, Achang, Bai, Naxi, Buyi, Lahu, Yi, Wa, and Han teachers; (3) geographic spread across multiple border prefectures and counties; (4) representation of both the primary and the secondary level; and (5) direct experience providing family education guidance to ethnic minority families. Consistent with grounded theory methodology, sampling proceeded iteratively: later participants were chosen in part to test and elaborate the categories emerging from earlier analysis, and recruitment continued until the research team judged that additional interviews were no longer generating new conceptual categories—the point of theoretical saturation (Corbin and Strauss, 2015). This sampling strategy aimed to explore and understand the family education guidance competency of rural teachers in ethnic minority border regions while comprehensively documenting the competencies, challenges, and professional development needs of teachers in this region regarding family education guidance.

Interviews were conducted in the language in which each participant was most comfortable—predominantly Mandarin Chinese, with participants free to code-switch into their ethnic mother tongue when conveying culturally specific concepts. Bilingual teachers themselves clarified ethnic-language terms where needed, and in cases where participants' Mandarin proficiency was limited, a bilingual community member known to the participant was present to facilitate communication. All interviews were audio-recorded with informed consent and transcribed verbatim in Chinese. Because the manuscript is written in English, a rigorous translation procedure was followed: all excerpts quoted in the text were translated into English by the bilingual research team; every quoted passage was then independently back-translated into Chinese and cross-checked by both authors against the original Chinese transcript to verify semantic equivalence; ethnic-language material embedded in the transcripts was verified with the participant or with a community member fluent in the relevant language; and where literal and idiomatic renderings diverged, the meaning of the Chinese original was treated as authoritative.

The interview protocol was designed to elicit participants' own experiences and perspectives rather than to test predefined categories. Its broad thematic focus drew on the domains identified in the literature review—the multidimensional nature of family education guidance competency, the difficulties teachers encounter in practice, and the conditions that support competency development—which served as sensitizing concepts orienting the inquiry; the questions themselves, however, were kept open-ended and non-leading so that the specific competency dimensions could emerge inductively from participants' accounts, in keeping with grounded theory. The protocol comprised eight primary questions; the complete interview protocol is provided in the Supplementary Material (Appendix A). These included items addressing the requirements of rural family education guidance (“What aspects of guidance do you believe rural family education requires?”), teachers' own guidance practice (“What family education guidance have you provided to students' parents?”), the difficulties they encountered (“What difficulties do you perceive in conducting family education guidance?”), and their views on the competencies the work demands (“What family education guidance competencies do you believe teachers should possess?”). The demographic characteristics of the interview participants are presented in Table 1.

**Table 1:** Demographic characteristics of interview participants.

No	Age	Gender	Ethnicity	Position	School
001	35	Female	Zhuang	Teacher	Panlong Primary School, Malipo County
002	33	Female	Hani	Teacher	Lianhuatan Township Central Primary School, Hekou County
003	45	Female	Yao	Teacher	Mengla Township Central Primary School, Jinping County
004	37	Female	Han	Teacher	Babu Middle School, Malipo County
005	45	Male	Wa	Teacher	Gasa Township Central Primary School, Jinghong City
006	30	Male	Miao	Teacher	Donglang Primary School, Malipo County
007	40	Male	Lisu	Teacher	Nuozhadu Township Middle School, Lancang County
008	46	Male	Dai	Teacher	Fengping Township Central Primary School, Mangshi
009	30	Female	Jingpo	Teacher	Jiele Primary School, Ruili City
010	35	Female	Achang	Teacher	Zhazi Village Primary School, Yingjiang County
011	28	Female	Bai	Teacher	Heshun Township Central Primary School, Tengchong County
012	45	Female	Naxi	Teacher	Gongshan Primary School, Tengchong County
013	50	Male	Miao	Teacher	Luosa Village Central School, Maguan County
014	28	Male	Buyi	Teacher	Laomeng Township Central Primary School, Jinping County
015	34	Male	Zhuang	Teacher	Donggan Middle School, Malipo County
016	36	Female	Lahu	Teacher	Menghan Township Middle School, Jinghong City
017	38	Female	Han	Teacher	Shilong Primary School, Malipo County
018	46	Male	Yi	Teacher	Huimin Township Central Primary School, Lancang County
019	50	Female	Dai	Teacher	Mukang Primary School, Mangshi
020	58	Male	Achang	Teacher	Nongdao Ethnic Middle School, Ruili City
021	49	Male	Dai	Teacher	Nongzhang Township Middle School, Yingjiang County
022	37	Female	Zhuang	Teacher	Naye Primary School, Funing County
023	36	Male	Yao	Teacher	Guichao Middle School, Funing County
024	42	Male	Han	Teacher	Datianba Primary School, Funing County
025	30	Female	Zhuang	Teacher	Anliang Primary School, Funing County

### 3.3 Data analysis procedures

Following established grounded theory methodology, the research team conducted systematic analytical procedures on the collected interview data:

**Code (Participant Quote / Open Code):** Initially, the researchers organized and assigned identification codes to the collected interview transcripts, utilizing identifiers such as “YMY-001” wherein the capitalized letters represent the interviewer's initials and “001” denotes the respondent number, thereby distinguishing interview content across different participants. Through intensive line-by-line analysis of these materials, multiple key concepts were identified and labeled—for example, “systematic knowledge mastery” and “understanding of local culture”—yielding a total of 108 discrete concepts. These concepts were subsequently integrated into 37 broader categorical groupings, such as “professional knowledge” and “cultural understanding.”

**Sub-Theme:** During this analytical phase, researchers conducted deeper exploration of the open codes, systematically identifying and labeling interrelationships among them. This process involved focusing on individual categories, examining their internal properties and external connections, and defining these relationships as axial categories. For instance, “solution provision” and “family education problem solving” were consolidated into the “problem-solving skills” axial category. Through this systematic approach, the research team constructed 11 axial categories.

**Theme:** In the final analytical phase, researchers identified key “core categories” demonstrating rich conceptual connections with the maximum number of axial categories. Centering on these core categories, a comprehensive theoretical framework was constructed. Through iterative comparative analysis, systematic screening, and progressive refinement, five core categories ultimately emerged Supplementary Material (Appendix B), providing the theoretical foundation for the research findings.

### 3.4 Trustworthiness

To ensure the trustworthiness of this grounded theory study, strategies aligned with the qualitative research paradigm were employed, focusing on credibility, dependability, confirmability, and transferability (Lincoln and Guba, 1985).

#### 3.4.1 Credibility

Several techniques were used to enhance the credibility (the qualitative parallel to internal validity) of the findings. First, triangulation was achieved through multiple means: data were collected from teachers across different ethnic minority communities (Yi, Zhuang, Hani, Dai, Wa, Jingpo, and Lahu) and different border counties; multiple researchers were involved in the coding process; and the findings were compared against the existing literature on family engagement in culturally diverse rural settings (e.g., Valdés, 1996; Burke, 2017). Second, prolonged engagement was established before formal data collection. The first author spent 3 months building rapport with participating schools and communities, which allowed teachers to become familiar with the researcher and to speak more openly about their guidance practices. Third, peer debriefing was conducted throughout the research process. Regular meetings were held with two doctoral colleagues who were not involved in the data collection; these peers reviewed the emerging codes and interpretations, challenged the researcher's assumptions, and provided critical feedback that helped refine the analysis. Fourth, following the derivation of preliminary categories and the theoretical model, the researchers consulted three peer experts (two university-based qualitative methodologists and one experienced rural teacher educator), who evaluated the coding scheme and the logical coherence of the emerging model. Modifications were made based on their recommendations.

#### 3.4.2 Researcher positionality and reflexivity

This study is grounded in a constructivist epistemology, which holds that knowledge is co-constructed between researchers and participants rather than discovered as an external reality. The researchers' own cultural proximity to the communities studied—both authors are based at Wenshan University in Yunnan Province and have worked extensively with ethnic minority schools in the border region—afforded valuable contextual understanding and facilitated rapport with participants. At the same time, this proximity carried the risk of importing taken-for-granted assumptions into the analysis. To make these influences transparent and analytically productive rather than invisible, reflexive memos were maintained throughout the research process. A concrete example illustrates how this practice shaped analytic decisions. During open coding, a recurring pattern was initially labeled “parental resistance to schooling.” A reflexive memo prompted the research team to recognize that this label imported a deficit-based assumption about minority parents and obscured what participants were actually describing—namely, a cautious stance rooted in families' historical experience of educational marginalization and in their rational assessment of economic returns to schooling in the border region. The code was accordingly relabelled “economically and historically grounded skepticism,” and this change altered the interpretation of the surrounding category: rather than framing the relevant teacher competency as a matter of overcoming parental deficiency, the analysis came to conceptualize it as the capacity to engage respectfully with a stance that families adopt on the basis of lived experience—a reframing that is reflected in the discussion of “perspective transformation” in Section 4.4.

#### 3.4.3 Dependability and confirmability

Dependability (the qualitative parallel to reliability) and confirmability (the qualitative parallel to objectivity) were addressed through an audit trail. Two researchers engaged in collaborative coding: one researcher executed primary coding tasks while the other conducted independent review of the coding results at each stage (open, axial, and selective coding). When initial coding yielded divergent interpretations, discrepancies were temporarily preserved and discussed in research team meetings until consensus was reached, with the goal of ensuring that the final codes and categories were grounded in the data rather than in the researchers' preconceptions. All coding decisions, memos, and records of team discussions were retained as part of the audit trail, allowing an external reviewer to trace the analytical process.

#### 3.4.4 Transferability

While generalizability is not a goal of grounded theory, thick description of the research context—including the characteristics of the 25 teacher participants, the 25 border counties of Yunnan Province, and the ethnic minority communities involved—is provided to allow readers to assess the transferability of the findings to other rural, ethnically diverse, or borderland settings.

## 4 Findings and discussion

Grounded in systematic application of grounded theory methodology, three-level coding of the raw interview data yielded five core categories: knowledge and professional competency, communication and interaction competency, cross-cultural and multilingual competency, guidance and motivation competency, and psychological support and management competency. The core concepts comprising these five modules demonstrate mutual influence, and the subordinate axial concepts similarly exhibit interactive relationships. The following sections provide detailed analysis of the meaning and connotations of each module and its constituent concepts, followed by comprehensive analysis of inter-category relationships, culminating in the construction of the competency model.

### 4.1 Analysis of the “knowledge and professional competency” core theme

“Knowledge and professional competency” in family education represents an essential competency for rural teachers in ethnic minority border regions engaged in family education guidance—a finding consistently emphasized across multiple studies examining teachers' family education guidance competency. Rural teachers must possess systematic family education theoretical knowledge and professional abilities, which facilitate more effective family education guidance work and enable the provision of scientifically-grounded support and guidance for students' holistic development. “Knowledge and professional competency” encompasses three subordinate categories: family education theoretical knowledge, family education research ability, and problem-solving ability (see Table 2).

**Table 2:** Coding examples for knowledge and professional competency.

Theme	Sub-theme	Code (participant quote/open code)
Knowledge and professional competency	Theoretical knowledge of family education	“*Teachers need to master systematic and professional family education knowledge” (Data-YWY-001)*
		“*Master the professional ethical norms and professional knowledge of family education guidance” (Data-XXP-003)*
	Research ability in family education	“*Rural family education generally has many problems, such as parents believing that education is only the responsibility of schools and teachers” (Data-ZXY-006)*
	Problem-solving ability	“*Teachers need to actively communicate with parents to solve communication difficulties between parents and children” (Data-WWS-001)*

#### 4.1.1 Family education theoretical knowledge

Family education theoretical knowledge constitutes the foundational skill enabling rural teachers to conduct effective family education guidance. Interview participants including teachers, school administrators, and educational experts unanimously affirmed that “teachers need to master systematic and professional family education knowledge,” encompassing knowledge of fundamental principles, theoretical frameworks, educational methods, and pedagogical techniques within family education. Crucially, in the context of Yunnan Province border regions, this theoretical knowledge must be adapted to address the specific family structures and childrearing norms of ethnic minority communities. For example, Teacher 003 (Yao ethnicity, Jinping County) explained that effective family education guidance in Yao communities requires understanding of the clan-based social structure in which child-rearing decisions are made collectively by extended family rather than by parents alone: “When I talk to parents about their child's learning, I have learned I must also consider the grandparents' and elders' views, because in our community they have the final word.” This insight illustrates how theoretical knowledge about family systems must be refracted through the lens of local ethnic cultural norms to be practically applicable.

#### 4.1.2 Family education research ability

Family education research ability represents an essential competency for rural teachers confronting specialized challenges and context-specific problems. Family education in rural areas presents distinctive circumstances and needs. Rural teachers must understand and systematically investigate the current status and existing problems within rural family education to provide appropriately targeted guidance and support for parents. In the Yunnan border context, this research ability must extend to understanding how border-crossing dynamics shape family life. Several participants described situations where families had relatives living across the international border in Myanmar, Laos, or Vietnam, and where educational decisions were influenced by cross-border economic opportunities and social networks. Teacher 007 (Lisu ethnicity, Lancang County) noted: “Many of my students' fathers work seasonally across the border. The family's attitude toward education changes depending on whether there is work available over there. If there is, they may see no point in children studying.” Effectively researching and responding to these complex, border-specific family education dynamics requires competencies that go well beyond what is described in general rural education literature.

#### 4.1.3 Problem-solving ability

Problem-solving ability constitutes a necessary competency enabling rural teachers to address parents' needs for family education guidance. The family education environment in rural areas is characterized by complexity and variability; consequently, rural teachers must possess flexibility and adaptability, demonstrating capacity for rapid response and effective problem resolution. In border minority communities, problem-solving takes on dimensions absent from mainstream contexts. A recurring challenge described by participants was navigating situations where parents' distrust of formal educational institutions—rooted in historical experiences of ethnic minority marginalization under state education policy—created barriers to engagement. Teacher 013 (Miao ethnicity, Maguan County) recounted:

“Some older parents had bad experiences when they were young, when the school punished children for speaking the Miao language. So now when I come to talk to them as a teacher, they do not trust me at first. I have to work slowly, showing I respect their language and their way of doing things, before they will listen.”

This example illustrates that problem-solving in this context is not simply a matter of communicative technique but requires historically and culturally informed sensitivity to longstanding tensions between ethnic minority communities and state educational institutions. This pattern is consistent with the scholarship on bilingual-education policy and the historical marginalization of ethnic languages in Chinese minority schooling reviewed in Section 2.3 (Wan and Liu, 2012; Teng, 2001), which documents how earlier phases of assimilationist language policy generated an enduring wariness between minority families and state schools that frontline teachers must actively work to repair.

### 4.2 Analysis of the “communication and interaction competency” core theme

Rural teachers must possess well-developed “communication and interaction competency,” which facilitates the establishment of positive relationships with parents, strengthens mutual understanding and trust, and thereby enables more effective family education guidance work. “Communication and interaction competency” encompasses two subordinate categories: interpersonal communication ability and home–school communication and cooperation ability (see Table 3).

**Table 3:** Coding examples for communication and interaction competency.

Theme	Sub-theme	Code (participant quote/open code)
Communication and interaction competency	Interpersonal communication ability	“*Teachers need to actively communicate with parents, share students' school situations with them and provide suggestions.” (Data-XXP-006)*
	Home-school communication and cooperation ability	“*The ability for teachers to establish good communication channels with parents and actively cooperate.” (Data-ZQF-005)*

#### 4.2.1 Interpersonal communication ability

Interpersonal communication ability represents a critical skill for rural teachers engaged in family education guidance. Rural teachers must possess sophisticated communication skills, demonstrating capacity to listen attentively to parents' perspectives and concerns, understand parents' expectations and needs, and articulate their own viewpoints and recommendations with clarity. In the Yunnan border context, interpersonal communication frequently requires navigating significant asymmetries of educational attainment and social status. Many ethnic minority parents in border counties have completed only primary school or are functionally illiterate in Mandarin, which means that communication must be conducted in plain, accessible language and often through oral rather than written channels. Teacher 006 (Miao ethnicity, Malipo County) described his adaptation of communication practices:

“I never send home a written letter because many parents cannot read Chinese well. I go to their home or I call them. And I speak slowly and use simple words. Sometimes I use our Miao language. That is when they really listen.”

This account illustrates that interpersonal communication competency in this setting is inseparable from cultural and linguistic sensitivity—it is not merely a matter of speaking clearly, but of choosing the right language, medium, and relational register for each family.

#### 4.2.2 Home-school communication and cooperation ability

Home-school communication and cooperation ability constitutes a key competency for rural teachers conducting family education guidance. Through sustained communication and cooperation with parents, rural teachers can more effectively understand students' family backgrounds, cultural practices, and educational needs, thereby enabling more appropriately targeted educational interventions. In Yunnan's border regions, establishing effective home–school cooperation channels faces distinctive obstacles arising from geographic remoteness, multilingual households, and the labor migration patterns that frequently take parents across international borders. Teacher 017 (Han ethnicity, Malipo County) described how she adapted her cooperation practices to these conditions:

“Many families live scattered across mountain villages with no road access. I cannot rely on a parents' meeting at school—some would need half a day just to arrive. So I organize small group meetings at the village head's house during market days when families come down from the mountains. I also keep a WeChat group for each village, and I post short voice messages because typing is hard for many parents.”

This account reveals that home–school cooperation ability in this setting goes beyond maintaining regular contact; it demands the capacity to design locally adapted communication architectures that work within the physical, cultural, and technological constraints of border communities—a dimension of cooperation competency largely absent from existing frameworks developed in accessible, urban settings.

### 4.3 Analysis of the “cross-cultural and multilingual competency” core theme

Rural teachers in ethnic minority border regions engaged in family education guidance must possess cross-cultural and multilingual competency to ensure effective communication and successful transmission of educational concepts. This competency encompasses two subordinate categories: cross-cultural communication ability and ethnic language proficiency (see Table 4).

**Table 4:** Coding examples for cross-cultural and multilingual competency.

Theme	Sub-theme	Code (participant quote/open code)
Cross-cultural and multilingual competency	Cross-cultural communication ability	“*Some parents in rural areas have lower educational levels and don't know how to help children with homework or educate children.” (Data-ZXY-003)*
		Ethnic language proficiency	“*Some ethnic minority parents can only understand ethnic minority languages and don't understand Mandarin.” (Data-WWS-001)*

#### 4.3.1 Cross-cultural communication ability

Cross-cultural communication ability represents an essential competency for rural teachers conducting family education guidance within specialized cultural contexts. Ethnic minority communities in border regions possess distinctive cultural backgrounds, and parents' cultural orientations and educational beliefs differ substantially from those prevalent in urban areas. Rural teachers must possess cross-cultural competency—specifically, the capacity to understand and respect family education differences across diverse cultural backgrounds. In Yunnan's border minority communities, this cross-cultural competency encompasses understanding of specific ethnic cultural practices around children, learning, and intergenerational relationships. For instance, in Dai communities (represented by participants 008, 019, and 021), learning is traditionally embedded within community and religious life, with Buddhist temple education having historically complemented or even superseded formal schooling. Teacher 019 (Dai ethnicity, Mangshi) noted:

“In Dai culture, a child going to the temple to learn from the monks is seen as a great honor. Some parents feel the state school takes their child away from this path. So I have learned to talk about how the two can work together, not compete.”

This illustrates how cross-cultural communication competency in this setting demands not merely cultural sensitivity in a generic sense, but deep, group-specific knowledge of how educational value is constructed within each ethnic community.

#### 4.3.2 Ethnic language proficiency

Ethnic language proficiency constitutes an important competency for rural teachers conducting family education guidance within specialized linguistic contexts. When rural teachers possess ethnic language abilities, they can communicate more effectively with ethnic minority parents, understand their needs and concerns, and provide more appropriately targeted family education guidance. The linguistic situation in Yunnan's border regions is exceptionally complex: a single school may serve families speaking Dai, Jingpo, Achang, and Mandarin, among other languages, and many first-generation ethnic minority parents have limited Mandarin proficiency. Several participants described the practical weight of this challenge: Teacher 009 (Jingpo ethnicity, Ruili City) recounted: “When I speak to a parent in Jingpo, their face changes. They relax. They say more. When I have to use Mandarin, they answer yes or no and look away. The language is the door.” This vivid account foregrounds how ethnic language proficiency is not merely a communication tool but a signal of cultural belonging and respect that fundamentally shapes whether parents feel safe enough to engage in genuine dialogue about their children's education. It also points to a significant challenge for teacher preparation programs: producing teachers who are not only pedagogically qualified but also functionally multilingual in the relevant local languages.

### 4.4 Analysis of the “guidance and motivation competency” core theme

Guidance and motivation competency is critically important for rural teachers in ethnic minority border regions engaged in family education guidance. Due to distinctive geographic circumstances and ethnic cultural variations, family education beliefs and practices may differ substantially from those in other regions. “Guidance and motivation competency” primarily encompasses two dimensions: perspective transformation and motivation to participate (see Table 5).

**Table 5:** Coding examples for guidance and motivation competency.

Theme	Sub-theme	Code (participant quote/open code)
Guidance and motivation competency	Perspective transformation	“*Rural family education concepts are relatively backward, need to change parents' concepts and let them realize that learning has a great impact on children.” (Data-WWS-006)*
	Motivation to participate	“*Rural teachers need to awaken parents' awareness of educational responsibility, cultivate their preliminary educational skills.” (Data-ZXY-003)*

#### 4.4.1 Perspective transformation

Interview findings revealed that “some parents in rural families are unwilling to allow their children to pursue education, believing that schooling is useless—this perspective requires transformation.” Rural teachers must guide parents toward transformed perspectives on family education through educational programming, parent meetings, and related initiatives, enabling parents to recognize the importance of family education. In the border minority context, perspective transformation is a particularly sensitive task because teachers must challenge deeply held cultural and economic beliefs without dismissing or delegitimizing the lived realities that produced them. In communities where agricultural or cross-border trade income can in the short term exceed what education offers, and where ethnic minority youth have historically faced discrimination in labor markets despite educational achievement, skepticism about schooling is not simply “backward”—it reflects a rational response to structural inequity. Teacher 022 (Zhuang ethnicity, Funing County) reflected:

“I used to go to parents and just tell them education is important for the future. But they would say, my brother never finished school and he earns more than any teacher. So now I try to understand their thinking first. I ask what they hope for their child. And then I find the connection between their hope and what school can offer. That is the real work.”

This account demonstrates that effective perspective transformation in this context requires a dialogic approach that begins with genuinely honoring parents' perspectives rather than attempting to replace them.

#### 4.4.2 Motivation to participate

Interview findings revealed that “many parents in rural families migrate for employment and rarely participate in family education.” Rural teachers must motivate parents to participate in family education activities through dissemination of family education resources and educational methods, thereby enhancing their educational competencies. In the border region context, motivating participation requires creative adaptation to the constraints of migrant labor patterns and geographic distance. Several participants described using mobile phone communication platforms—particularly WeChat, which has high penetration even in remote rural communities (China Internet Network Information Center, 2024)—to maintain contact with absent parents and to share brief video or audio-based educational guidance. Teacher 020 (Achang ethnicity, Ruili City) described his practice:

“I make short recordings in Mandarin and in Achang and send them in the class group. I tell the parents what their child did well this week and what they can try at home. The fathers who are far away working can listen when they have time. Some of them reply. They feel they are still part of things.”

This approach—adapting family education guidance to the realities of physical absence through bilingual digital outreach—represents a distinctively border-region form of the participation motivation competency that has no direct equivalent in existing Western or urban Chinese literature.

### 4.5 Analysis of the “psychological support and management competency” core theme

Rural teachers in ethnic minority border regions engaged in family education guidance must possess psychological support and management competency. Given the specialized challenges confronting family education—including limited educational backgrounds and significant family pressures—rural teachers must provide emotional support to parents and assist them in problem resolution. “Psychological support and management competency” encompasses two subordinate categories: psychological counseling ability and organizational management ability (see Table 6).

**Table 6:** Coding examples for psychological support and management competency.

Theme	Sub-theme	Code (participant quote/open code)
Psychological support and management competency	Psychological counseling ability	“*Rural teachers also need to help parents reduce emotionalization and change parents' educational mentality.” (Data-WWS-003)*
	Organizational management ability	“*Rural teachers need to have organizational, coordination, and communication abilities to effectively carry out family education guidance work.” (Data-ZZX-003)*

#### 4.5.1 Psychological counseling ability

Family education in ethnic minority border regions may confront distinctive psychological challenges, including mental health issues among left-behind children and parental stress and anxiety. Rural teachers must possess psychological support ability—specifically, the capacity to understand and attend to the psychological needs of parents and students. In Yunnan's border communities, the psychological challenges are compounded by a specific constellation of stressors: the grief and anxiety of left-behind children separated from migrant parents; the burden on elderly grandparent caregivers managing households without spousal support; and the social stigma that ethnic minority families sometimes experience in relation to Mandarin-language schooling. Teacher 011 (Bai ethnicity, Tengchong City) described the emotional texture of her guidance work:

“One child in my class cried every day for the first month of school because his mother had gone to work in Kunming. The grandmother came to me in tears. She did not know what to do. I spent more time with her than with any other parent that year—not just talking about school, but listening to how hard it was for her. Only after she felt heard did she trust me enough to work with me on the child.”

This account illustrates that psychological counseling ability in this context must extend beyond technically supporting parents' educational practices to encompassing empathic relational support for the emotional realities of border community family life.

#### 4.5.2 Organizational management ability

Rural teachers must allocate time judiciously, maintaining appropriate balance between teaching responsibilities and family education guidance work. Teachers should demonstrate capacity to reasonably schedule classroom instruction and home visits, ensuring balance between these domains. In border region schools, organizational management competency must also encompass the ability to coordinate across multi-ethnic community structures. Teacher 018 (Yi ethnicity, Lancang County) noted:

“In our area we have Yi families, Lahu families, and Han families all in the same school. When I organize a parent activity, I have to think about language—can I get someone to translate? I have to think about the date—does it conflict with an ethnic festival? I have to think about the place—is it accessible for families who live far up in the mountains? Each of these things requires planning that teachers in cities probably never think about.”

This testimony underscores that organizational management in this context is inseparable from cultural coordination competency, making it qualitatively distinct from the management skills addressed in general teacher competency frameworks.

### 4.6 The family education guidance competency model

The family education guidance competency model for rural teachers in ethnic minority border regions is constructed upon the foundation of inter-category relationships. This model was developed to address the distinctive educational environment characteristic of ethnic minority border regions and to respond to the specialized needs of rural teachers engaged in family education guidance.

Knowledge and professional competency, together with communication and interaction competency, constitute essential competencies for teachers conducting family education guidance. Knowledge and professional competency provides teachers with foundational support in family education theoretical knowledge and pedagogical expertise. Communication and interaction competency establishes the foundation for effective teacher-parent communication.

Cross-cultural and multilingual competency represents a key competency within the specialized context of ethnic minority border regions. Teachers must demonstrate capacity to understand and respect families from diverse cultural backgrounds and to communicate with parents across multiple languages.

**Figure 1:**
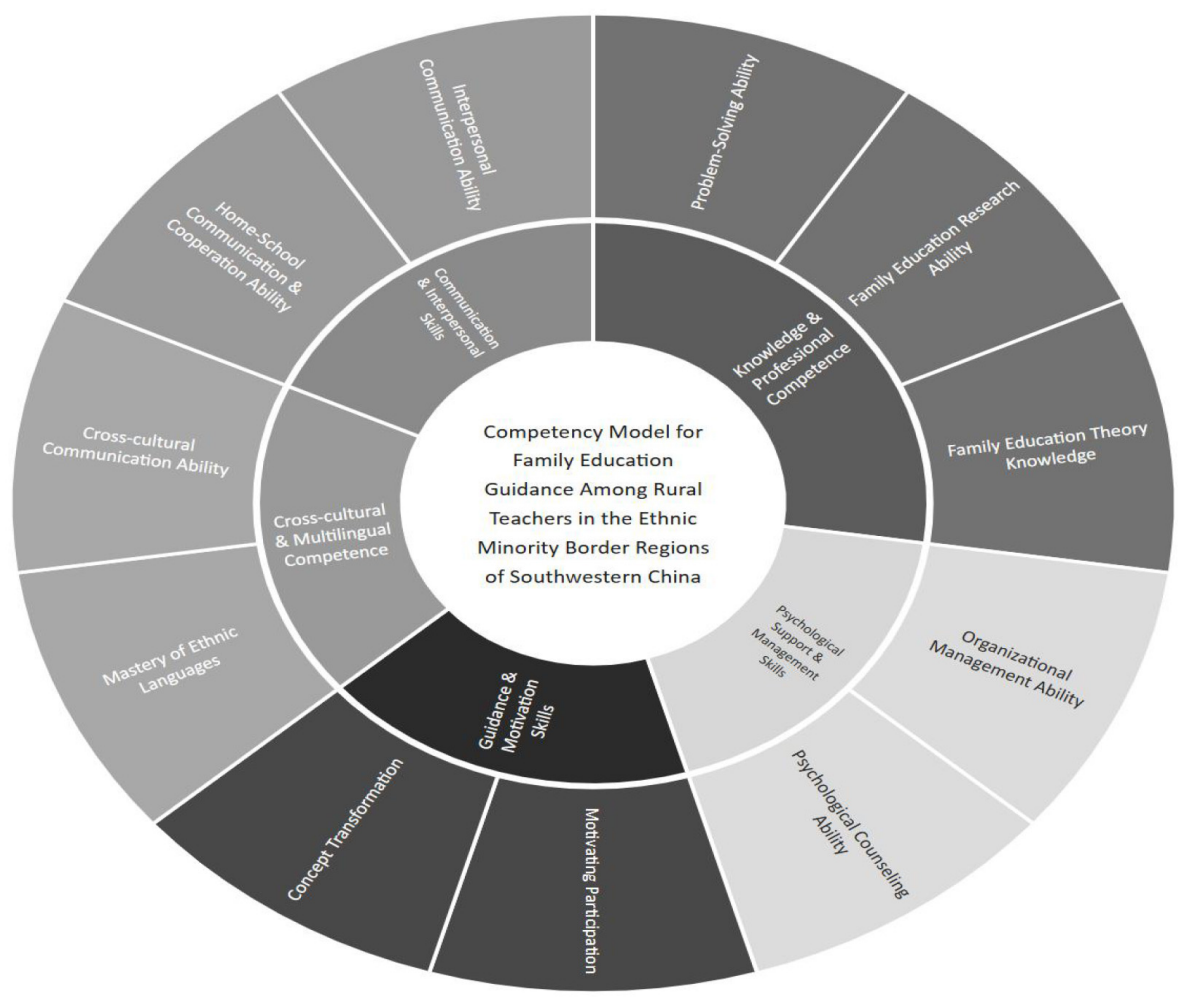
Family education guidance competency model for rural teachers in ethnic minority border regions of Southwestern China (Yunnan Province).

Guidance and motivation competency, together with psychological support and management competency, constitute core competencies essential for achieving family education guidance objectives. These competencies are systematically interrelated, collectively constituting the family education guidance competency model for rural teachers (see Figure 1).

## 5 Conclusions and recommendations

This study employed a grounded theory approach to construct a family education guidance competency model for rural teachers in ethnic minority border regions. Specifically, the model draws on semi-structured interviews with 25 primary and secondary school teachers from fifteen ethnic groups, working in rural schools distributed across the border prefectures and counties of Yunnan Province in southwestern China—a frontier region that adjoins Myanmar, Laos, and Vietnam and is home to more than twenty long-settled ethnic minority groups. Grounded in the lived experiences, voices, and interview accounts of these teachers, the model comprises five interrelated core categories—knowledge and professional competency, communication and interaction competency, cross-cultural and multilingual competency, guidance and motivation competency, and psychological support and management competency—and offers a systematic, context-sensitive account of what it takes for these teachers to bridge the divide between school and home. Based on this competency structure, the following recommendations are proposed.

First, strengthen teacher training and professional development. Systematic professional training programs targeting rural teachers in ethnic minority border regions should be designed and implemented, with emphasis on cultivating family education guidance competency. This encompasses conducting specialized courses, seminars, and workshops, as well as providing individualized instructional support and mentoring. For example, preparation that is participatory, experiential, and case-based, rather than lecture-driven, has been shown to be more effective in building teachers' capacity for family–school partnership work (Willemse et al., 2016). In the border-region context, such training could be organized around locally sourced case studies, for instance simulated home visits conducted partly in an ethnic language, or guided practice in supporting the grandparent caregivers of left-behind children.

Second, establish supportive platforms and professional networks. Dedicated online platforms should be created to provide rural teachers with relevant resources, information, and opportunities for professional exchange regarding family education guidance. Such platforms can facilitate experience sharing and mutual assistance while enabling timely resolution of challenges encountered during family education guidance implementation. Evidence from resource-constrained rural primary schools in Gansu indicates that school-based teacher professional learning communities and teaching-research groups can connect professional development to teachers' everyday classroom realities even where material resources are limited (Sargent and Hannum, 2009). An online platform for border-region teachers might accordingly be built around such peer learning communities, allowing teachers in geographically isolated schools to circulate locally effective practices, such as bilingual voice-message outreach to migrant parents, across county and prefecture boundaries.

Third, strengthen interdisciplinary collaboration. Experts from diverse fields, educational institutions, and community organizations should be encouraged to participate collaboratively in rural teachers' family education guidance work. Through interdisciplinary collaboration, the needs of rural students and families can be addressed more comprehensively, providing more effective support and services. The action-team model for school, family, and community partnerships, in which a standing team coordinates the contributions of educators, families, and community members, offers one tested structure for organizing this collaboration (Epstein, 2011; Henderson and Mapp, 2002). In border communities, such a team could be deliberately composed to include village heads, bilingual community members, and, where appropriate, the temple or clan elders whom several participants identified as holding decisive influence over children's education.

Fourth, emphasize reflective practice. Rural teachers should be encouraged to integrate theoretical knowledge with practical experience, continuously enhancing their family education guidance competency through professional practice. Simultaneously, reflection mechanisms should be established to facilitate timely synthesis of experiential insights, enabling continuous improvement and refinement of guidance methods and strategies. The reflexive practice that informed the present study illustrates the value of this habit: a reflexive memo led the research team to relabel an initially deficit-framed code, “parental resistance to schooling,” as “economically and historically grounded skepticism,” which in turn reframed the associated competency from overcoming parental deficiency to engaging respectfully with families' lived experience. A comparable practice of documenting and interrogating one's own assumptions can help frontline teachers recognize when a family's caution reflects rational experience rather than indifference, and adjust their guidance accordingly.

These recommendations, together with the illustrative examples that accompany them, are offered as starting points rather than prescriptions. Because each is derived from one specific border context, it must be adapted to the actual wants, needs, and circumstances of the particular ethnic minority border community in which it is applied, and developed in consultation with the families and communities it is intended to serve.

Taken together, these competencies and recommendations carry significance that extends beyond the model itself. Rural teachers in ethnic minority border regions stand on the frontline of educational equity, frequently serving children and families who face compounding linguistic, cultural, geographic, and socioeconomic barriers—conditions under which family involvement is consistently associated with children's academic and social development (Fan and Chen, 2001; El Nokali et al., 2010). By naming and structuring the competencies these teachers require, the study reframes family education guidance from an implicit expectation into a teachable, assessable, and improvable set of capabilities, offering a concrete foundation for training, professional development, and policy design. In doing so, it builds on and extends established frameworks of parental involvement and engagement (Epstein, 2011; Goodall and Montgomery, 2014) by foregrounding the cross-cultural and multilingual realities that are central to ethnic minority border regions yet receive comparatively little attention in frameworks developed largely in Western, monolingual contexts; it thereby responds to recent calls to examine teachers' readiness for parental engagement across diverse policy and cultural settings (Antony-Newman, 2024). Because the model is grounded in the accounts of teachers working in one specific context, these competencies necessarily reflect that setting, and the extent to which they transfer to other regions or education systems remains an empirical question. Future research should therefore further deepen and empirically test these competencies across diverse settings. Ultimately, strengthening the family education guidance competency of rural teachers is not a peripheral concern but a meaningful lever for change—one that reaches the wellbeing of children, the resilience of families, and the long-term development of border and ethnic minority communities. We hope this work advances both scholarly understanding and frontline practice, and that it serves as a catalyst for sustained investment in the very teachers who, quietly and persistently, help hold these communities' educational futures in their hands.

## Data Availability

The raw data supporting the conclusions of this article will be made available by the authors, without undue reservation.

## Footnotes

1 In this article, ethnic minority border regions refer to areas located along a nation's land frontiers where ethnic minority groups constitute a substantial share of the population, marked by geographic remoteness and cultural-linguistic plurality. In the present study, the term specifically denotes the 25 border counties of Yunnan Province in southwestern China, which adjoin Myanmar, Laos, and Vietnam and are home to more than twenty long-settled ethnic minority groups.
