# Supplementary material for: Constructing a competency model for family education guidance among rural teachers in ethnic minority border regions: a grounded theory approach
Source: Front Psychol. 2026 Jul 6;17:1772630. doi: 10.3389/fpsyg.2026.1772630 (PMC13381232; doi:10.3389/fpsyg.2026.1772630)
Supplement: Supplementary file 1 [file Supplementary_file_1.docx]

# Appendix A. Semi-Structured Interview Protocol

The following protocol guided the semi-structured interviews. Each interview was conducted in the language in which the participant was most comfortable; participants were free to code-switch into their ethnic mother tongue, and follow-up probes were used to elicit concrete examples and clarify culturally specific terms. The eight primary questions were:

1. What aspects of guidance do you believe rural family education requires?

2. What family education guidance have you provided to students’ parents?

3. What difficulties do you perceive in conducting family education guidance?

4. What family education guidance competencies do you believe teachers should possess?

5. When working with families from different ethnic groups, what language or languages do you use, and how do you proceed when parents are more comfortable in an ethnic mother tongue than in Mandarin?

6. Can you describe a specific instance in which you communicated with a parent or guardian—for example, a grandparent caring for a left-behind child—about a student’s learning or development?

7. What knowledge, skills, or institutional support do you feel you still need in order to guide families more effectively in this region?

8. In what ways do the history, culture, and economic circumstances of your community shape how families view schooling, and how do you respond to this in your guidance work?

# Appendix B. Codebook: From Concepts to Core Categories

Open coding of the 25 interview transcripts generated 108 discrete concepts, which were consolidated into 37 concept categories, then into 11 axial categories, and finally into 5 core categories. The table below summarizes the resulting three-level coding structure, with a representative emic indicator for each axial category drawn from participants’ own accounts.

| **Core Category (Selective Coding)** | **Axial Category** | **Representative Emic Indicator** |
| --- | --- | --- |
| **Knowledge and Professional Competency** | Family Education Theoretical Knowledge | Systematic mastery of family-education theory and the professional and ethical norms of guidance |
|  | Family Education Research Ability | Diagnosing locally recurrent problems, such as parents viewing education as solely the school’s responsibility |
|  | Problem-Solving Ability | Proactively resolving parent–child and home–school communication difficulties |
| **Communication and Interaction Competency** | Interpersonal Communication Ability | Sharing a student’s school situation with parents and offering concrete suggestions |
|  | Home-School Communication and Cooperation Ability | Establishing durable communication channels and sustaining active cooperation with families |
| **Cross-cultural and Multilingual Competency** | Cross-cultural Communication Ability | Adapting guidance for parents with limited schooling and differing cultural expectations |
|  | Ethnic Language Proficiency | Using or mediating ethnic mother tongues when parents have limited command of Mandarin |
| **Guidance and Motivation Competency** | Perspective Transformation | Shifting parents’ beliefs about the value and long-term impact of schooling |
|  | Motivation to Participate | Awakening parents’ sense of educational responsibility and basic guidance skills |
| **Psychological Support and Management Competency** | Psychological Counseling Ability | Reducing parental emotionality and helping reshape the educational mindset |
|  | Organizational Management Ability | Organizing, coordinating, and managing family-education guidance activities |

Note. Core and axial category labels are reproduced verbatim from the selective- and axial-coding results reported in Section 4; representative indicators are condensed paraphrases of the open-coding concepts and participant statements presented in Tables 2–6. Data sources are identified by anonymized participant codes (e.g., Data-YWY-001).
